# Polyurethanes Synthesized with Blends of Polyester and Polycarbonate Polyols—New Evidence Supporting the Dynamic Non-Covalent Exchange Mechanism of Intrinsic Self-Healing at 20 °C

**DOI:** 10.3390/polym16202881

**Published:** 2024-10-12

**Authors:** Yuliet Paez-Amieva, Noemí Mateo-Oliveras, José Miguel Martín-Martínez

**Affiliations:** Adhesion and Adhesives Laboratory, University of Alicante, 03080 Alicante, Spain; yuliet.paez@ua.es (Y.P.-A.); noemi.mateo@ua.es (N.M.-O.)

**Keywords:** intrinsic self-healing at 20 °C, polyurethane, polyol blends, polycarbonate soft segments, polyester soft segments, mechanism of self-healing

## Abstract

Polyurethanes (PUs) synthesized with blends of polycarbonate and polyester polyols (CD+PEs) showed intrinsic self-healing at 20 °C. The decrease in the polycarbonate soft segments content increased the self-healing time and reduced the kinetics of self-healing of the PUs. The percentage of C-O species decreased and the ones of C-N and C=O species increased by increasing the polyester soft segments in the PUs, due to higher micro-phase separation. All PUs synthetized with CD+PE blends exhibited free carbonate species and interactions between the polycarbonate and polyester soft segments to a somewhat similar extent in all PUs. By increasing the polyester soft segments content, the storage moduli of the PUs decreased and the tan delta values increased, which resulted in favored polycarbonate soft segments interactions, and this was related to slower kinetics of self-healing at 20 °C. Although the PU made with a mixture of 20 wt.% CD and 80 wt.% PE showed cold crystallization and important crystallinity of the soft segments, as well as high storage moduli, the intercalation of a small amount of polycarbonate soft segments disturbed the interactions between the polyester soft segments, so it exhibited self-healing at 20 °C. The self-healing of the PUs was attributed to the physical interactions between polycarbonate soft segments themselves and with polyester soft segments, and, to a minor extent, to the mobility of the polymeric chains. Finally, the PUs made with 40 wt.% or more polyester polyol showed acceptable mechanical properties.

## 1. Introduction

The synthesis of polyurethanes (PUs) is carried out by addition reactions of polyols and polyisocyanates. Their structure consists of soft segments or domains (long polyol chains) and hard segments or domains (urethane and/or urea bonds) [1]. Different polyols can be used in the synthesis of PUs, mainly polyesters, polyethers, and polycarbonates. The use of polyester polyols results in PUs with good mechanical properties and chemical resistance but insufficient hydrolytic degradation. However, the use of polycarbonate polyols results in PUs with excellent resistance to hydrolytic degradation, heat, and weathering [2]. Therefore, the optimal properties of PUs cannot be reached with only one polyol, and balanced properties can be obtained by mixing polyols of a different nature. In fact, the mixture of polyester and polyether polyols in the synthesis of waterborne polyurethanes (PUDs) increased crystallinity and improved mechanical properties [3,4]. Similarly, improved properties were obtained in PUDs synthesized with mixtures of polycaprolactone and polyether polyols [5] and polyester and polycarbonate polyols [6].

The physical interactions between the soft segments determine the properties of PUs and PUDs. These interactions become more complex when the soft segments have a different chemical nature. Recently, it has been shown that the mixture of polyester and polycarbonate polyols changed the extent and type of interactions between them, more noticeably when they contained less than 50 wt.% polyester [7]. The different structural features of the mixtures of polyester and polycarbonate polyols were due to the disturbance of carbonate–carbonate interactions and the formation of new ester–carbonate and hydroxyl–carbonate interactions. Furthermore, ester–carbonate and carbonate–carbonate interactions explained the self-adhesion of the mixtures of polyols.

Currently, the development of self-healing polymers, mainly polyurethanes, is of great interest [8]. Because of the hydrogen bonds (i.e., non-covalent dynamic bonds) between urethane and/or urea groups in PUs, micro-phase separation is produced, and this may favor self-healing [9,10]. Several PUs exhibited self-healing induced by an extrinsic (isocyanate encapsulation) [11] or an intrinsic (no need for an external trigger input) [12,13] mechanism.

Self-healing PUs are currently used in leather coatings, photo-luminescent materials, flexible electronic components, and biomaterials. Waterborne PU coatings containing disulfide bonds on leather require 12 h to self-repair at 60 °C [14], and PUs containing polyimine for flexible electronics require 24 h to self-repair at 30 °C [15]. Ying et al. [16] synthesized a waterproof and self-healing PU intended for electronic skins. Self-healing PUs based on dynamic aromatic disulfide bonds have been developed for biomedical applications and they required 48 h to complete self-healing [17]. In turn, Jiang et al. obtained biocompatible and biodegradable polyoxime PU elastomers that show spontaneous self-healing in a physiological environment [18].

Although polyester or polyether polyols are commonly used to synthesize self-healing PUs [2,19], self-healing is favored when polycarbonates are used [20,21,22]. Thus, the self-healing of PUs made with polycarbonate and containing amide moieties is due to amide–carbonate hydrogen bonds [23]. Furthermore, PUs synthesized with poly(hexamethylene) carbonate diol exhibited self-healing after heating at 120 °C for 1–7 h [24] or upon heating at 80 °C for 40 s [25]. On the other hand, PUs made with blends of polycarbonate and propylene glycol polyols exhibited self-healing after heating at 37 °C for 6 h, and it was due to hydrogen bonding and flexible and short mobile polymeric chains [26].

**Figure 1:**
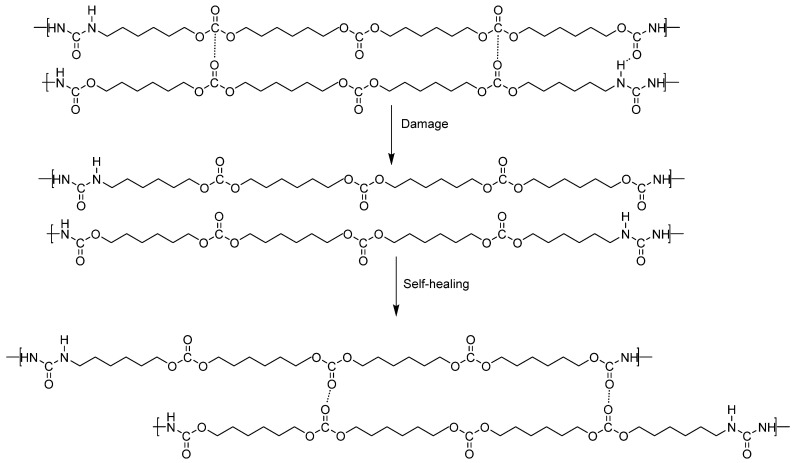
Proposed mechanism of self-healing at 20 °C in YCD polyurethane based on dynamic non-covalent exchange between polycarbonate soft segments. Adapted from reference [27].

Most of the already synthesized self-healing PUs require temperature and/or a long time to completely self-heal at ambient temperature. In our recent study [27], a low-hard-segment-content (22 wt.%) PU made with polycarbonate of 1,6-hexanediol—YCD—showed intrinsic self-healing at 20 °C. The self-healing of YCD was due to a significant number of free carbonate groups, a low percentage of urethane groups, and the high mobility of the soft segments. The dynamic non-covalent exchange between polycarbonate soft segments was proposed as the mechanism of self-healing (Figure 1).

To confirm the mechanism of self-healing depicted in Figure 1, in this study, different PUs with a similarly low hard segment content were synthesized with mixtures of polycarbonate of 1,6-hexanediol and polyadipate of 1,6-hexanediol. The mixture of the polyols reduces the percentage of polycarbonate soft segments in the PUs, and, if the interactions among polycarbonate soft segments favor self-healing at 20 °C, the extent of self-healing of the PUs will decrease.

Because the mechanical properties of YCD were low and those of the PU made with polyester polyol (YPE) were high [27], PUs synthetized with blends of polycarbonate of 1,6-hexanediol and polyadipate of 1,6-hexanediol may exhibit improved mechanical properties. Therefore, in this study, the self-healing, chemical, structural, rheological, and mechanical properties of different PUs made with blends of polycarbonate of 1,6-hexanediol and polyadipate of 1,6-hexanediol were assessed to provide additional evidence of the proposed mechanism of self-healing (dynamic non-covalent exchange between polycarbonate soft segments).

## 2. Materials and Methods

### 2.1. Materials

The PUs were obtained by reacting 4,4′ methylene bis (cyclohexyl) isocyanate (HMDI) (90% purity, Sigma Aldrich Co., St. Louis, MO, USA) with 1,4-butanediol (BD) chain extender (99% purity, Panreac Applichem^®^, Darmstadt, Germany), and two polyols with a molecular weight of 1000 Da: Polycarbonate of 1,6-hexanediol—CD—(Covestro, Leverkusen, Germany) and polyadipate of 1,6-hexanediol—PE (Synthesia, Barcelona, Spain).

### 2.2. Methods

#### 2.2.1. Synthesis of the Polyurethanes (PUs)

The NCO/OH ratio was 1.1, and the one-shot method was used for synthesizing the PUs. The polyols or polyol mixtures and 1,4-butanediol were heated at 80 °C inside a closed bottle and mixed in a double centrifuge SpeedMixer DAC 150.1 FVZ-K (FlackTek Inc., Landrum, SC, USA) at 2400 rpm for 1 min. After heating again at 80 °C for 10 min, the isocyanate was added to the polyols + 1,4-butanediol mixture and mixed in SpeedMixer equipment at 2400 rpm for 1 min. The curing of the PUs was carried out in an oven by heating gradually from 50 °C to 70 °C in half-hour cycles followed by curing at 80 °C for 6 h. After 24 h at room temperature, the PUs were annealed at 85 °C for 1 h. All PUs had a similar hard segment content (22–23 wt.%).

The nomenclature of the PUs made with CD+PE mixtures is “YxCDyPE” (“x” is the weight percentage of the CD polyol and “y” is the one of the PE polyol). Thus, Y2CD8PE is the PU synthesized with 20 wt.% polycarbonate of 1,6-hexanediol and 80 wt.% polyadipate of 1,6-hexanediol.

#### 2.2.2. Experimental Techniques

**Figure 2:**
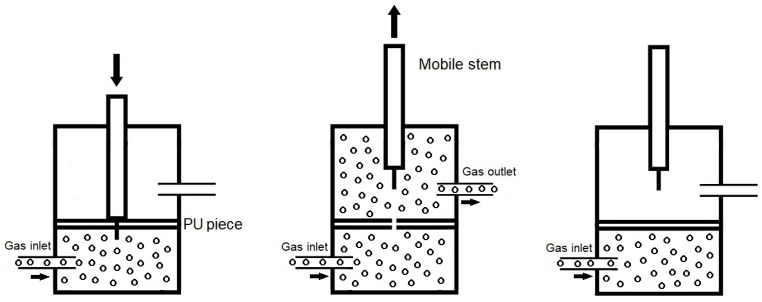
Diagram of the equipment used to assess the self-healing of the PUs.

Self-healing assessment: The equipment described in a previous study [28] was used to quantitatively assess the PUs' self-healing at 20 °C. In the middle of a hermetically closed chamber, a PU piece (19 mm diameter and 3 mm thick) was located (Figure 2) and pierced with a needle with a diameter of 1 mm. Upon withdrawing the needle, a nitrogen flow at a pressure of 750 mbar and a flow rate of 8 mL/min was allowed to flow upwards from the bottom until the gas flow on the top was null. The kinetics of self-healing was determined by the variation of the gas flow over time.

ATR-IR spectroscopy: The chemistry of the polyols blends and the PUs as well as the extent of micro-phase separation in the PUs were assessed by infrared spectroscopy in attenuated total reflectance mode. An Alpha spectrometer (Bruker Optik GmbH, Ettlinger, Germany) provided with a germanium prism was used. The angle of incidence of the IR beam was 45°, and 60 scans were performed with a resolution of 4 cm^−1^.

X-ray photoelectron spectroscopy (XPS): The chemistry on the PU surfaces were determined by XPS. A K-ALPHA instrument (Thermo Fisher Scientific, Waltham, MA, USA) provided with a hemispherical analyzer was used. Both survey scans (pass energy = 200 eV) and high-resolution C1s, O1s and N1s spectra (pass energy = 50 eV) were recorded.

Differential scanning calorimetry (DSC): The structural and thermal properties and the extent of micro-phase separation of the PUs were assessed using DSC Q100 equipment (TA Instruments, New Castle, DE, USA). A nitrogen flow rate of 100 mL/min and aluminum crucibles were used. The PU was heated from −80 °C to 200 °C at a heating rate of 10 °C/min. Then, the PU was cooled down to −80 °C (cooling rate = 10 °C/min), and, finally, a second heating run from −80 °C to 250 °C was carried out (heating rate = 10 °C/min).

X-ray diffraction (XRD): The crystallinity of the polyols blends and the PUs was assessed using Bruker D8-Advance equipment (Bruker, Etlinger, Germany) provided with a Kristalloflex K 760-80F X-ray generator (voltage: 20–60 kV; power: 3000 W; current: 5–80 mA) equipped with an X-ray tube with a copper anode. The incident X-ray beam had a wavelength of 1.54 nm. All PU samples had similar geometrical dimensions (diameter: 2 mm; thickness: 0.5 mm), and a parallel beam geometry for thin films was used. The X-ray diffractograms were obtained by using an angular rate of 0.05° every 3 s, and they were normalized to the maximum peak by using Evaluation 14.0.0.0 (2017) software.

Thermal gravimetric analysis (TGA): The variation in the weight loss of the polyols blends and the PUs as a function of the temperature (35 °C to 600 °C) was recorded using TGA Q500 equipment (TA Instruments, New Castle, DE, USA) by using a heating rate of 10 °C/min. The experiments were carried out in nitrogen (flow rate: 50 mL/min) and 9–10 mg sample placed in platinum crucible was used.

Dynamic mechanical thermal analysis (DMA): The viscoelastic properties of the PUs were measured using a DMA Q800 instrument (TA Instruments, New Castle, DE, USA). Samples with dimensions of 33 mm × 12 mm × 2.5 mm were used. The experiments were carried out at a frequency of 1 Hz in the single cantilever mode. The samples were heated from –100 °C to 80 °C at a heating rate of 5 °C/min.

Stress–strain test: Stress–strain tests were used to assess the mechanical properties of the PUs. The ASTM D 638-14 standard [29] was selected, and type 2 dumbbell specimens were used. The strain–stress tests were carried out using a Zwick/Roell Z005 universal testing machine (Barcelona, Spain) by using a pull-off rate of 100 mm/min. Three replicates for each PU were obtained and averaged.

## 3. Results

### 3.1. Assessment of PUs Self-Healing at 20 °C

The self-healing ability of PUs is commonly assessed by mechanical tests [30,31]. These tests are labor-intensive, they are not reproducible, and the kinetics of self-healing cannot be determined. For these reasons, new equipment and procedure for self-healing assessment have recently been developed [28]. This procedure is fast and reproducible, repetitive self-healing assessment is feasible, and the kinetics of self-healing can be accurately determined. Therefore, this procedure was used in this study.

The self-healing at 20 °C of PUs made with blends of polycarbonate of 1,6-hexanediol and polyadipate of 1,6-hexanediol (CD+PE) was qualitatively assessed by cutting a round PU piece with scissors and immediately joining the separated parts by hand. Video S1 shows as typical example the full process for Y6CD4PE. After 30 s of joining the two separated parts of Y6CD4PE, they self-healed.

**Figure 3:**
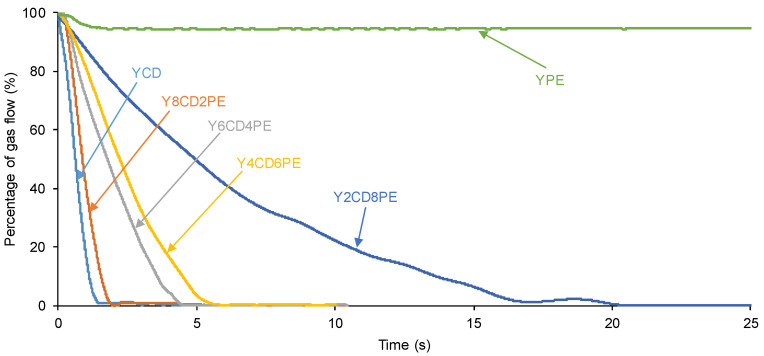
Kinetics of self-healing at 20 °C of PUs made with CD+PE blends.

**Figure 4:**
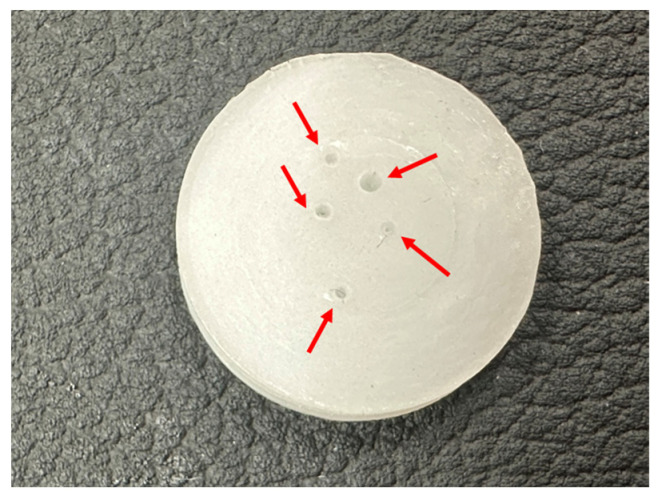
A photo of Y2CD8PE after the assessment of self-healing at 20 °C. The red arrows show the location of the punctures made during the experiment.

In order to quantitatively assess the self-healing at 20 °C of the PUs made with CD+PE blends, the equipment shown in Figure 2 was used. All PUs showed self-healing at 20 °C, except YPE, which does not contain polycarbonate soft segments (Figure 3). Figure 4 shows, as a representative example, the appearance of a Y2CD8PE piece after self-healing measurement.

**Figure 5:**
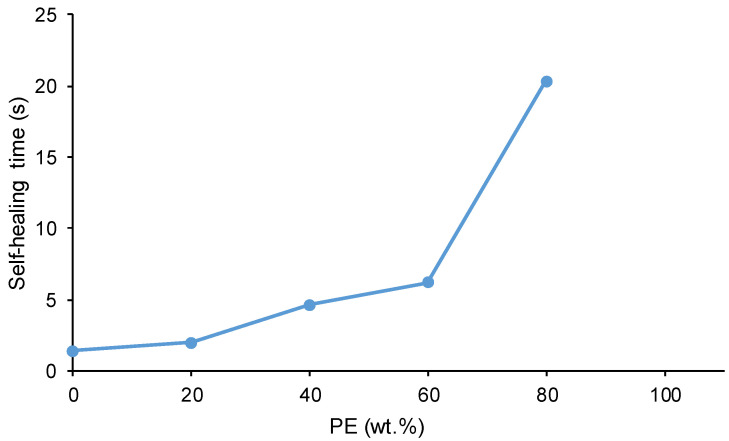
Variation of the self-healing time at 20 °C of the PUs made with CD+PE blends as a function of their PE content.

Figure 5 shows that the self-healing time at 20 °C of the PUs increases by decreasing their polycarbonate soft segments. The kinetics and time (1.4–2.0 s) of self-healing at 20 °C of YCD and Y8CD2PE are very similar (Figure 3 and Figure 5). The kinetics of self-healing at 20 °C are slower and the self-healing time (4.6–6.2 s) is longer in Y6CD4PE and Y4CD6PE; they are somewhat similar between them. The lesser self-healing ability of Y6CD4PE and Y4CD6PE can be ascribed to a significant decrease in polycarbonate soft segments. On the other hand, Y2CD8PE shows a long self-healing time at 20 °C (20.3 s), and the kinetics of self-healing is significantly longer than in the other PUs. Therefore, a small percentage of polycarbonate soft segments in Y2CD8PE seems sufficient to impart self-healing at 20 °C.

Because the variation in the gas flow vs. time of the self-healing curves of Figure 3 is exponential, they were fitted to first-order kinetics—Equations (1) and (2):

Rate = k [gas flow](1)

ln [gas flow] = −kt + ln [gas flow]o(2)

The plots of ln [gas flow] vs. time of the PUs made with CD+PE blends and their fitting to first-order kinetics are given in Figure S1 of the Supplementary Material file. The slopes of the linear plots of ln [gas flow] vs. time are the rate constants, and they are given in Table 1. The rate constants of the PUs made with CD+PE blends vary between 0.91 s^−1^ in YCD and 0.14 s^−1^ in Y2CD8PE, and they decrease steadily by increasing the polyester soft segments content in the PUs. Thus, the kinetics of self-healing becomes slower by decreasing the polycarbonate soft segments in the PUs.

### 3.2. Chemical Compositions of the PUs

In this study, the chemical compositions of the PUs made with CD+PE blends have been assessed by ATR-IR spectroscopy and XPS.

**Figure 6:**
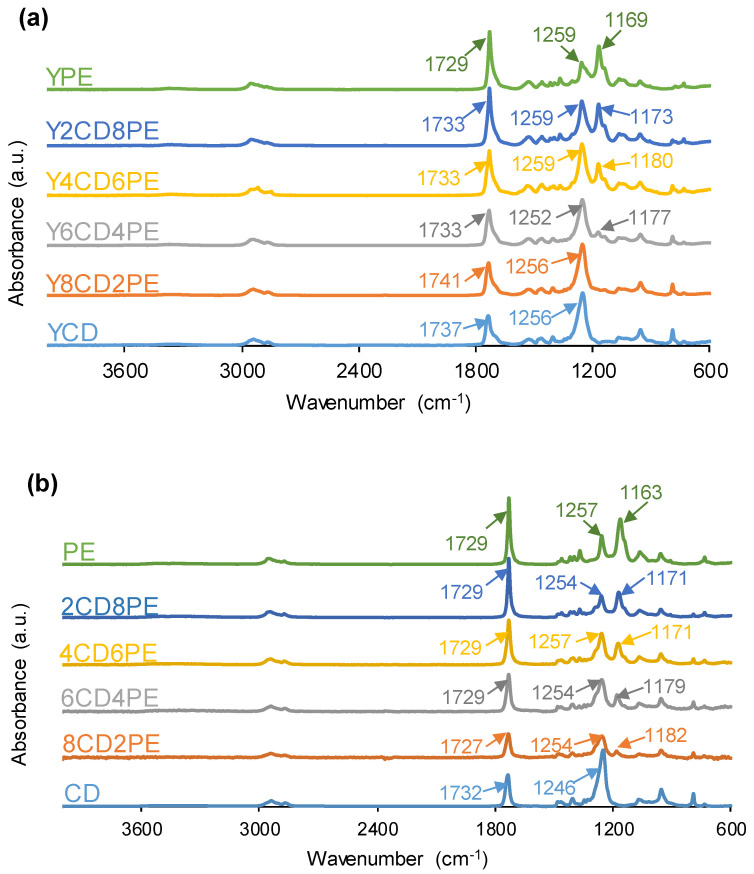
ATR-IR spectra of (**a**) PUs made with CD+PE blends and (**b**) CD+PE polyol blends.

Figure 6 shows the ATR-IR spectra of the PUs. All ATR-IR spectra exhibit similar absorption bands, but they differ in the OCC wavenumbers of the soft segments (1256–1259 cm^−1^ in polycarbonate soft segments and 1169–1180 cm^−1^ in polyester soft segments). The ATR-IR spectra of the PUs show the absorption bands of the hard segments—3356–3372 cm^−1^ (N-H stretching) and 1729–1741 cm^−1^ (C=O stretching of urethane/ urea)—and the soft segments—2938–2957 and 2863–2875 cm^−1^ (C-H stretching), 1465 and 1346–1370 cm^−1^ (C-H bending), and 1252–1259 and 1169–1180 cm^−1^ (C-O stretching). The wavenumbers of the IR bands were assigned according to previous research [32,33]. On the other hand, the polyol blends show the same absorption bands as the PUs (Figure 6) because of the low hard-segment contents of the PUs.

**Figure 7:**
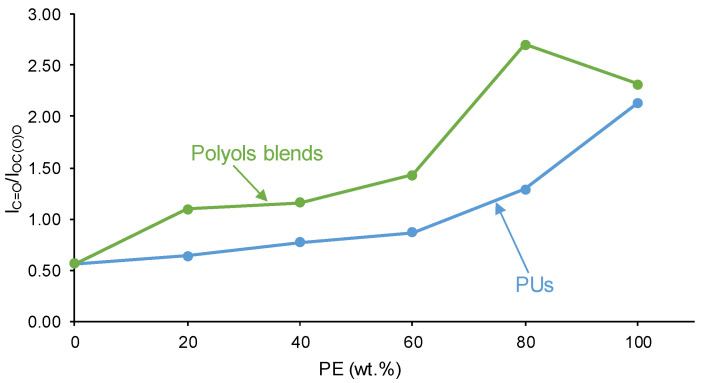
Variation of I_C=O_/I_OC(O)O_ ratios of PUs made with CD+PE blends and CD+PE polyol blends as a function of their PE content.

Because YPE has a higher number of ester groups (13) in the soft segments than carbonate groups (18) in the ones of YCD, the intensity of the C=O stretching band in the ATR-IR spectrum of YPE is the highest and the one of YCD is the lowest among all PUs (Figure 6). The increase in the polyester soft segments increases the I_C=O_/I_OC(O)O_ ratio—C=O band intensity/OC(O)O band intensity—in the ATR-IR spectra of the PUs (Figure 7), and the increase is more noticeable in the PUs made with more than 60 wt.% PE. YCD and Y8CD2PE show similarly small I_C=O_/I_OC(O)O_ ratios (0.5), indicating similar chemical structure, and this ratio is somewhat similarly high in Y4CD6PE and Y6CD4PE due to the almost similar contents of polycarbonate and polyester soft segments. It is interesting that the PUs showing fast kinetics of self-healing (Figure 3) also show lower I_C=O_/I_OC(O)O_ ratios in the ATR-IR spectra; this may indicate the relevance of polycarbonate soft segment interactions to self-healing at 20 °C. On the other hand, the trends of the I_C=O_/I_OC(O)O_ ratios of the PUs and the polyols blends as a function of their PE content are similar (Figure 7); however, the I_C=O_/I_OC(O)O_ ratios in the CD+PE polyol blends are higher than in their respective PUs. This can be ascribed to the urethane and urea groups and the stronger interactions between the soft segments in the PUs than among the polymeric chains of the polyol blends.

The interactions between organic carbonate groups have scarcely been studied in the existing literature. In 2001, Wang and Balbuena applied the ab initio and density functional theory methods to analyze the interactions between linear carbonate molecules of different nature [34]. These authors proposed that the dimers of carbonate molecules associated mainly via C-H···O intermolecular interactions, and that the interactions between similar carbonate molecule dimers were less stable than among carbonate molecules of different nature. Therefore, the existence of carbonate–carbonate interactions has been demonstrated elsewhere.

The curve fitting of the C=O stretching region of the ATR-IR spectra allows the assessment of different chemical species in the PUs. The individual carbonyl peaks were fitted by using a Gaussian function which, after background subtraction, was free for intensity and full width at half maximum (FWMH). The wavelengths of the different C=O species in the PUs were assigned according to previous literature [7,27,32,35,36]. Thus, the free C=O in polycarbonate was set at 1740 cm^−1^, the free C=O in polyester at 1730 cm^−1^, the free urethane at 1730 cm^−1^, the carbonyl–carbonyl interactions between the soft segments and the hydrogen-bonded carbonyl groups between hard and soft segments at 1712 cm^−1^, the free urea at 1699 cm^−1^, and the hydrogen-bonded urea at 1660 cm^−1^. Even amines were not used in the synthesis of the PUs, because their curing was carried out in an oven under air, urea groups were formed.

To verify the nature of the hydrogen-bonded carbonyl group in the PUs, CD and PE polyols were separately immersed in ammonia solution. The formation of hydrogen bonds between the N-H group of the ammonia solution and the C=O groups can be anticipated [36]. The ATR-IR spectra of the polyols immersed in ammonia solution show a new band at 1645–1646 cm^−1^ due to the formation of those hydrogen bonds.

**Figure 8:**
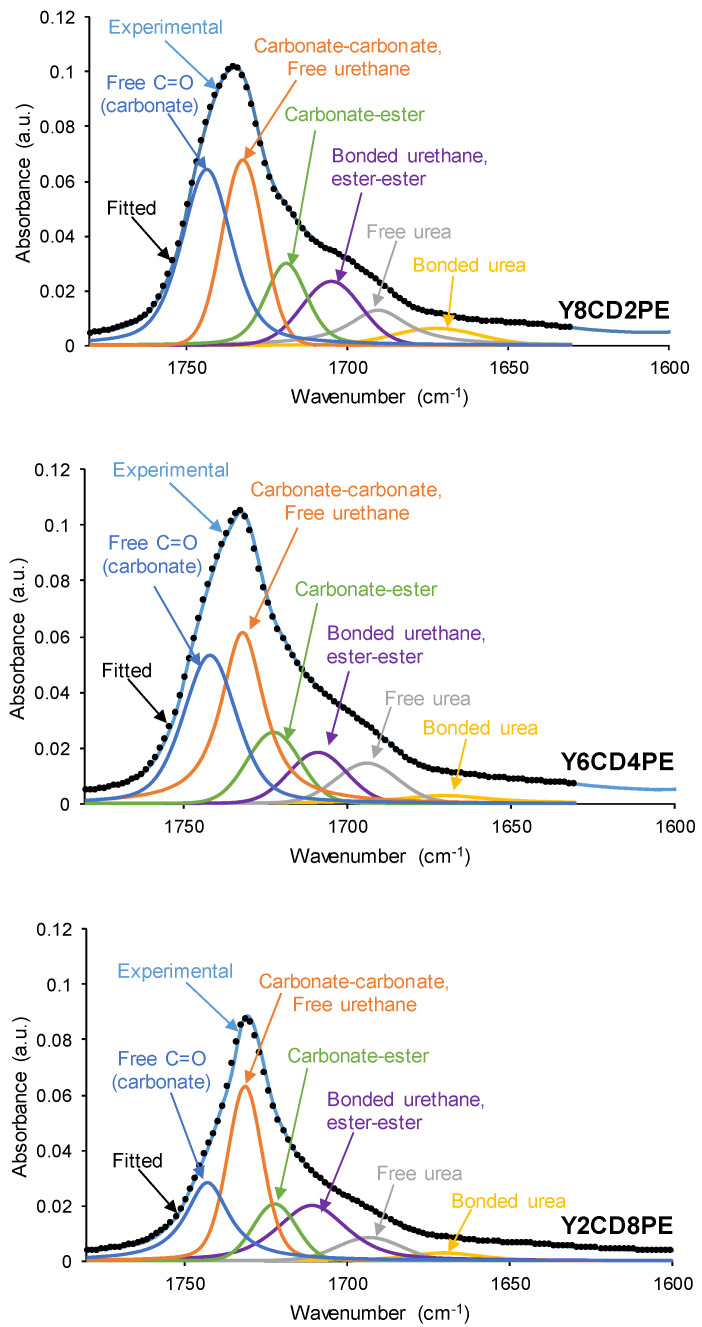
Curve fitting of the C=O stretching region of the ATR-IR spectra of some PUs made with CD+PE blends.

Figure 8 and Figure S2 of the Supplementary Material file show the curve fittings of the C=O stretching regions of the ATR-IR spectra of the PUs. Whereas 4–5 different C=O species (free and bonded urethane, free and bonded urea, and—only in YCD—free carbonate) can be distinguished in YCD and YPE, all PUs made with CD+PE blends show one additional contribution at 1720–1722 cm^−1^ due to carbonate–ester interactions between the soft segments [7] (Table 2). The percentages of these interactions are somewhat similar (10–13%) in PUs with different polyester soft segment content, even in the PU made with only 20 wt.% CD. On the other hand, the most abundant C=O species in PUs made with CD+PE blends are free urethane and free carbonate (Table 2).

Y8CD2PE shows less free urethane/carbonate–carbonate interactions and more free carbonate species than YCD because the addition of 20 wt.% PE during synthesis disrupts the interactions between the polycarbonate soft segments. Y4CD6PE and Y6CD4PE have similar free urethane/carbonate–carbonate interactions (31–35%) and free carbonate (31%) species; they differ in the higher percentage of hydrogen bonded urethane/ester–ester species and lower percentage of carbonate–ester species in Y4CD6PE (Figure 8, Table 2). Therefore, it seems that there is a competition between the polyester and polycarbonate soft segments to interact among them in Y4CD6PE and Y6CD4PE. Such competition can be associated with their somewhat similar self-healing time and kinetics of self-healing at 20 °C. The percentages of C=O species in Y2CD8PE differ significantly from the ones in the other PUs made with CD+PE blends (Figure 8, Table 2) because it has a lower percentage of free urethane/carbonate–carbonate interactions (23%) and higher percentage of hydrogen-bonded urethane/ester–ester interactions (23%). Thus, the mobility of the soft segments in Y2CD8PE should be partially restricted, and this can be related to its longer self-healing time and slower kinetics of self-healing. On the other hand, Y2CD8PE has 11% carbonate–ester interactions and 23% free carbonate species that are not present in YPE, and the percentage of free urethane species is significantly lower than in YPE (Table 2). Therefore, the existence of interactions between the polycarbonate and polyester soft segments (i.e., carbonate–ester) and free carbonate species in Y2CD8PE facilitates the mobility of polymeric chains and favors self-healing.

The degree of micro-phase separation in PUs is related to the occurrence of hydrogen bonding in the hard phase. It has been suggested elsewhere [36] that the degree of micro-phase separation in PUs can be derived from ATR-IR spectra by using the parameters determined by using Equations (3)–(7):

(3) $$X_{b}=\frac{A_{b}}{k’A_{f}+A_{b}}$$

(4) $$W_{h}=\frac{1-X_{b}f}{\left[1-X_{b}f+1-f\right]}$$

(5) $$MP=fW_{h}$$

(6) SP=MP+(1−f)

(7) HP=1−SP

where *f* is the hard segments molar ratio; *X_b_* is the hydrogen-bonded urethane group fraction; *W_h_* is the fraction of the hard segment dispersed in the soft segment; and *MP*, *SP*, and *HP* are the mixed-phase fraction, the soft-phase fraction, and the hard-phase fraction, respectively. On the other hand, *A_b_* is the absorbance of hydrogen-bonded urethane at 1707–1716 cm^−1^, *A_f_* is the absorbance of free urethane at 1730–1734 cm^−1^, and *k′* is a constant related to the ratio of the absorption coefficients of hydrogen-bonded and free urethane groups. In this study, the value of *k′* was taken as 1.2 [37].

According to Table 3, a similar *W_h_* fraction (0.15–0.19) is obtained in all PUs because they have similar fraction of the rigid phase dispersed in the soft phase. The values of *MP* (0.03–0.04), *SP* (0.81–0.82), and *HP* (0.18–0.19) are practically similar in all PUs. These results agree well with those previously reported in PUs with well-separated hard and soft domains [36,38].

A relatively important fraction of hydrogen-bonded urethane groups (*X_b_* = 0.19–0.37) is obtained in all PUs (Table 3). The lower the *X_b_* value, the lower the micro-phase separation. According to Table 3, PUs made with CD+PE blends exhibit higher micro-phase separation (*X_b_* = 0.29–0.37) than the ones made with one polyol only (*X_b_* = 0.23–0.27), and the micro-phase separation is more noticeable by increasing the PE content (Y6CD4PE is an exception—*X_b_* = 0.19). Therefore, more net interactions among the hard and the soft segments are obtained in PUs made with CD+PE blends than in the ones made with one polyol only, likely due to the interactions between polycarbonate and polyester soft segments. In Y6CD4PE, due to the competition between the polycarbonate and polyester soft segments to interact each other, significantly lower micro-phase separation is obtained than in the other PUs, and this may be associated with the higher movement of the polymeric chains.

XPS was used to assess the surface chemistry of the PUs. The chemical elements in all PU surfaces are carbon, oxygen, and nitrogen (Table 4). YCD and Y8CD2PE surfaces have almost similar carbon and oxygen atomic percentages, but the one of nitrogen is higher in Y8CD2PE due to higher micro-phase separation than in YCD. On the other hand, Y2CD8PE shows a lower atomic percentage of carbon and a higher atomic percentage of nitrogen than YPE. The most important differences in the chemical compositions correspond to the PUs made with 40–60 wt.% CD. Y6CD4PE shows the highest amount of carbon and the lowest amount of oxygen among all PUs, and it also has a lower atomic percentage of nitrogen than Y4CD6PE (Table 4). This trend agrees well with the lower micro-phase separation of Y6CD4PE evidenced in its ATR-IR spectrum and can be ascribed to the competition of the polycarbonate and polyester soft segments to interact between them and with the urethane/urea groups. On the other hand, the amount of nitrogen increases by decreasing the polycarbonate soft segments in the PUs because of the increase in micro-phase separation.

**Figure 9:**
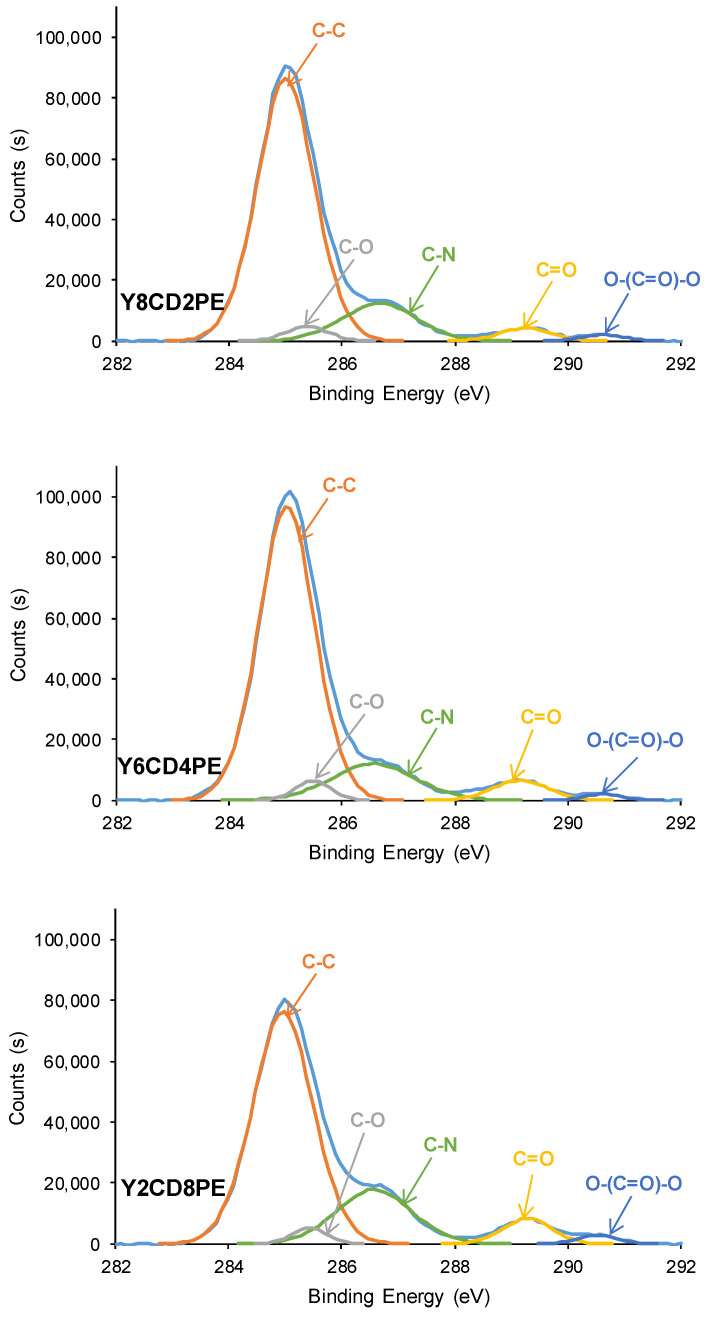
C1s photopeaks of some PUs made with CD+PE blend surfaces. XPS experiments.

The nature of the chemical species on PU surfaces was assessed by the curve fitting of the high-resolution C1s photopeaks. Five different chemical species (C-C, C-O, C-N, C=O, and O-(C=O)-O) can be distinguished in all PU surfaces, except in YPE (Figure 9 and Figure S3 of Supplementary Material file, Table 5).

The atomic percentages of C-C (binding energy = 284.9 eV) and C-O (binding energy = 285.4–285.6 eV) species in the PUs decrease by increasing the amount of polyester soft segments, although Y4CD6PE is an exception (Table 5). On the other hand, the atomic percentages of C-N (binding energy = 286.6–286.9 eV) and C=O (binding energy = 289.1–289.3 eV) species show the opposite trend, i.e., they increase by increasing the amount of polyester soft segments in the PUs, because of the increase in urethane/urea and ester species (Table 5). Y4CD6PE and Y2CD8PE have the highest percentages of C-N species among all PUs because of their higher content of urethane/urea groups and higher micro-phase separation. This agrees well with the trends of the ATR-IR spectra.

All PUs made with CD+PE blends and YCD show O-(C=O)-O species (binding energy = 290.5–290.8 eV) due to carbonate groups in the soft segments and, in general, their atomic percentages increase slightly by increasing the polyester soft segment content (Table 5). Interestingly, all PUs made with CD+PE blends have a higher atomic percentage (1.2–2.4 at.%) of O-(C=O)-O species than YCD (0.7 at.%), particularly Y4CD6PE and Y2CD8PE. This may indicate that the existence of polyester soft segments causes the disruption of the interactions between the polycarbonate soft segments in YCD, in agreement with our previous findings [7]. In PUs made with CD+PE blends, the increase in the polyester soft segments favors interactions with polycarbonate soft segments, resulting in a more ordered structure and less mobility of the polymeric chains, and this may be related to slower kinetics of self-healing at 20 °C.

### 3.3. Structural Properties of the PUs

The structural properties of the PUs were assessed by DSC, X-ray diffraction, TGA, and DMA.

**Figure 10:**
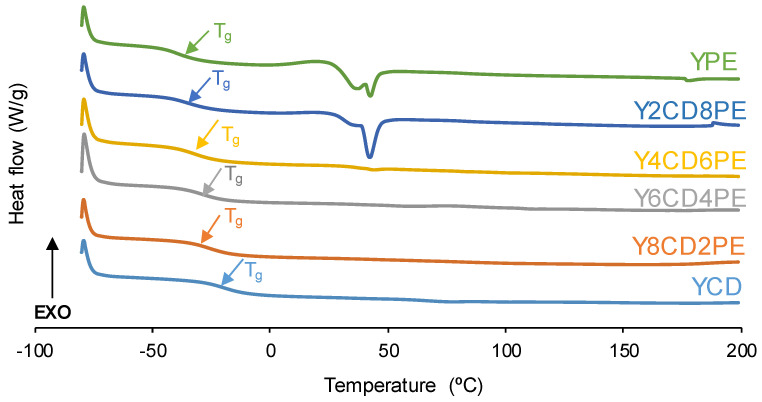
DSC curves of the PUs made with CD+PE blends. First heating run.

The DSC curves (first heating run) of all PUs (Figure 10) show the glass transition temperature (T_g_) of the soft segments (−21–−40 °C), and, in Y2CD8PE and YPE only, a small cold crystallization at 21–22 °C followed by the melting of the polyester soft segments at 42 °C can be distinguished. The T_g_ values of the PUs decline gradually by increasing their polyester soft segments content because of the stronger interactions between polycarbonate soft segments than between polyester soft segments (Table 6). The heat capacity at constant pressure (∆c_p_) in the glass transition of the PUs is related to the interactions between soft segments. Whereas YCD shows the lowest ∆c_p_ value, the PUs made with 40–60 wt.% PE show the highest ∆c_p_ values because of the existence of stronger interactions between the soft segments than in YCD, i.e., the intercalation of polyester soft segments among polycarbonate soft segments. This agrees well with the interactions between the polyester and polycarbonate soft segments evidenced by ATR-IR and XPS, as well as with the somewhat fast kinetics of self-healing at 20 °C in the PUs made with 40–60 wt.% PE. Furthermore, Y8CD2PE shows a higher ∆c_p_ value than YCD because the presence of a few polyester soft segments increases the interactions with the polycarbonate soft segments.

On the other hand, Y2CD8PE and YPE exhibit low T_g_ values, cold crystallization, and melting of the polyester soft segments [24] (Figure 10), but the ∆c_p_ value is significantly lower and the enthalpies of the cold crystallization and the melting of the soft segments are higher in YPE (Table 6). Therefore, YPE has a highly ordered structure of the soft phase due to high micro-phase separation [29] and cannot exhibit self-healing at 20 °C. However, even Y2CD8PE shows cold crystallization of the soft segments. The presence of a few polycarbonate soft segments is sufficient to disrupt the interactions between the polyester soft segments and increase the mobility of the polymeric chains, so self-healing at 20 °C is produced.

To remove the thermal history of the PUs, after cooling down to −80 °C, a second DSC heating run was performed. The DSC curves of the second heating run (Figure S4 of Supplementary Material file) show only two glass transitions due to the soft (T_ss_: −18–−38 °C) and hard (T_hs_: 236–241 °C) segments. The T_ss_ values of the PUs decrease and the T_hs_ values increase by increasing their polyester soft segment content (Table 7). This evidences higher micro-phase separation, which restricts the movement of the polymeric chains. The same evidence was obtained by ATR-IR spectroscopy. As a consequence, by increasing the polyester soft segments content in PUs made with CD+PE blends, slower kinetics of self-healing can be expected (Figure 3).

**Figure 11:**
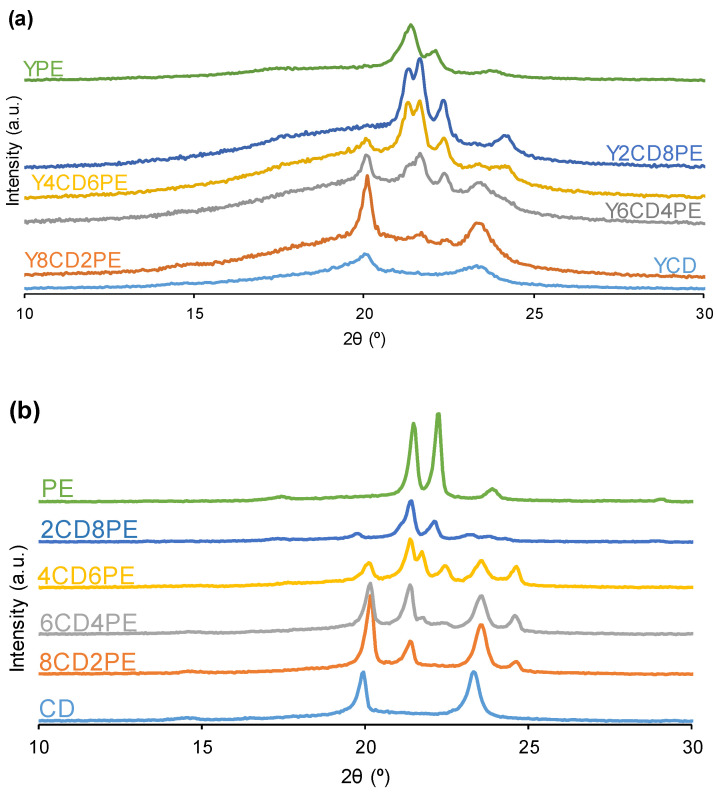
X-ray diffractograms of (**a**) PUs made with CD+PE blends and (**b**) CD+PE blends.

All PUs are semi-crystalline because they show different diffraction peaks in their X-ray diffractograms (Figure 11). Whereas the polycarbonate soft segments of YCD exhibit diffraction peaks at 2θ = 20.1° and 2θ = 23.4°, the polyester soft segments of YPE show diffraction peaks at 2θ = 21.4° and 2θ = 22.1° [27]. All PUs made with CD+PE blends show the diffraction peaks of both polycarbonate and polyester soft segments (Figure 11, Table S1 of Supplementary Material file) and also one new additional diffraction peak at 2θ = 21.5–21.8°; this peak is due to a mix phase produced by interactions between the polyester and polycarbonate soft-segments. In fact, the diffraction peak at 2θ = 21.5–21.8° is not present in individual polyols nor in their blends (Figure 11). Furthermore, less intense and broader diffraction peaks of all PUs than the ones of their corresponding polyols blends are noticed, and the higher the polyester soft segments content, the less intense and broader the diffraction peaks of the PUs are (Figure 11).

**Figure 12:**
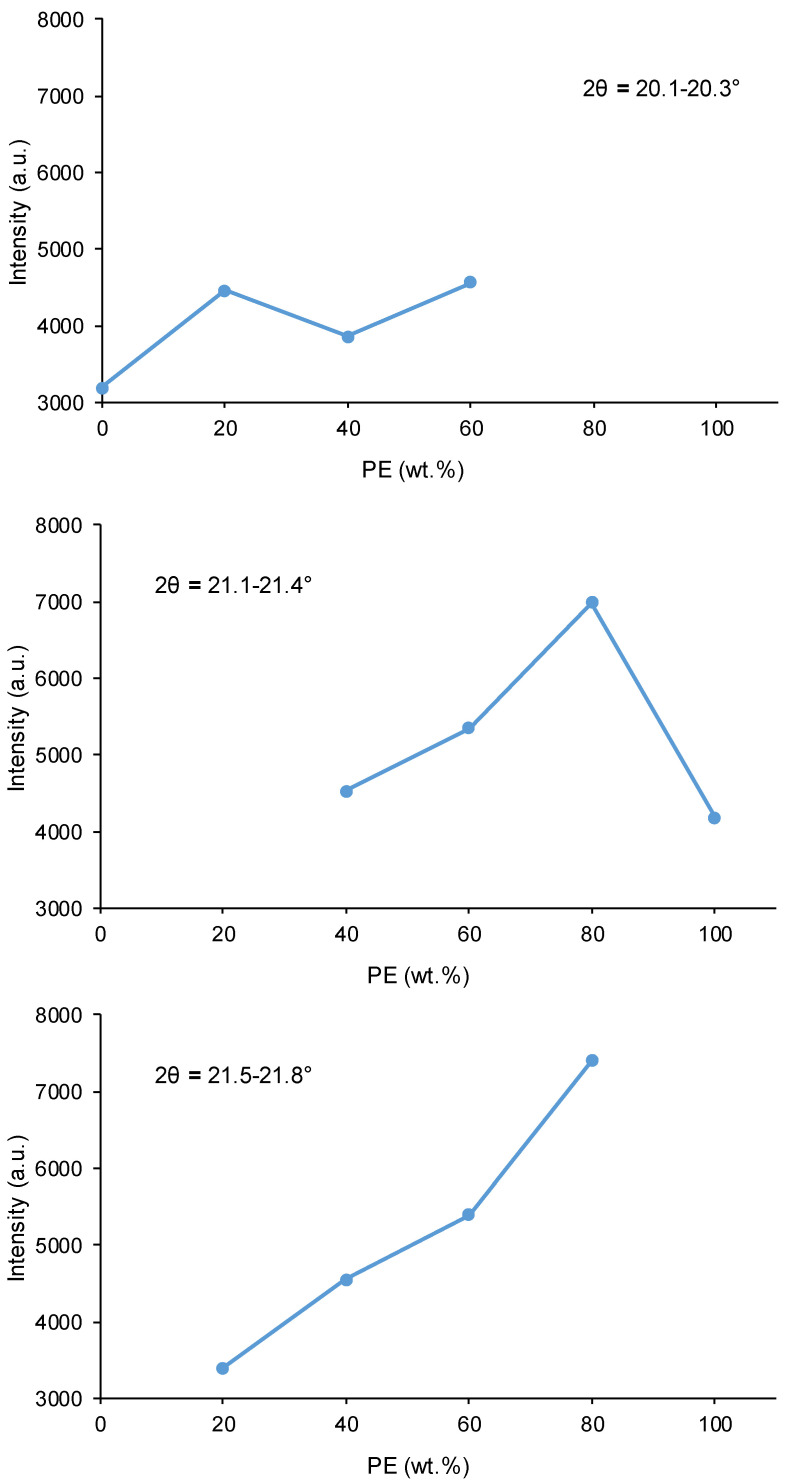
Variation in the intensities of different X-ray diffraction peaks of the PUs made with CD+PE blends as a function of their PE content.

According to Figure 12, in PUs made with CD+PE blends, the diffraction peak at 2θ = 21.5–21.8° becomes more intense by increasing their polyester soft segments content; i.e., the mix phase becomes more relevant. At the same time, the intensity of the diffraction peak of the polycarbonate soft segments at 2θ = 20.1–20.3° is higher in Y8CD2PE than in YCD, and it does not vary by increasing the polyester soft segment content. Furthermore, in PUs made with CD+PE blends, the diffraction peak of the polyester soft segments at 2θ = 21.1–21.4° is more intense by increasing the polyester soft segments content (Figure 12), and its intensity is higher than in YPE.

The different trends of the diffraction peaks of the PUs (Figure 12) are indicative of three distinctive structures:

- The diffraction peak of the polycarbonate soft segments at 2θ = 20.3° in Y8CD2PE is more intense than the one in YCD, and a new peak at 2θ = 21.8° due to polyester soft segments–polycarbonate soft segments interactions appears; furthermore, the diffraction peaks of the polyester soft segments are absent. Therefore, in Y8CD2PE, the few polyester soft segments are intercalated between the dominant polycarbonate soft segments, which results in higher crystallinity. The mixture of 80 wt.% CD + 20 wt.% PE polyols shows a similar X-ray diffractogram to Y8CD2PE, although the diffraction peaks are less intense in Y8CD2PE (Figure 11). Thus, the interactions between polycarbonate soft segments are dominant in Y8CD2PE, similarly to YCD, so both PUs show somewhat similar short self-healing times and fast kinetics of self-healing at 20 °C (Figure 3).
- The X-ray diffractograms of Y6CD4PE and Y4CD6PE show similarly intense peaks of polycarbonate soft segments at 2θ = 20.1–20.3°, the peaks of the polyester soft segments at 2θ = 21.1–21.4° (more intense in Y4CD6PE), and additional peaks at 2θ = 21.5–21.8° due to polyester soft segments–polycarbonate soft segments interactions (more intense in Y4CD6PE). Therefore, in Y6CD4PE and Y4CD6PE, the crystallinities due to each kind of soft segments can be distinguished, as well as the one due to the interactions between soft segments of different nature; this indicates the intercalation of the polycarbonate and polyester soft segments. Because the interactions between the polycarbonate soft segments are less intense than the ones among the polycarbonate and polyester soft segments, the self-healing times are longer and the kinetics of self-healing at 20 °C of Y6CD4PE and Y4CD6PE are slower than in YCD and Y8CD2PE (Figure 3).
- The X-ray diffractogram of Y2CD8PE shows the most intense peak of the polyester soft segments at 2θ = 21.4° and the one of the polyester soft segments–polycarbonate soft segments interactions at 2θ = 21.7°; the diffraction peaks of the polycarbonate soft segments are absent. Therefore, the structure of Y2CD8PE is dominated by the interactions between the polyester soft segments with some contribution of the interactions between polyester and polycarbonate soft segments. Thus, the polycarbonate soft segments are intercalated between the dominant polyester soft segments, enhancing the crystallinity of Y2CD8CD with respect to YPE. YPE and Y2CD8PE have somewhat similar structures, but, due to the polycarbonate soft segments–polyester soft segments interactions, Y2CD8PE is the only one exhibiting self-healing at 20 °C (Figure 3).

**Figure 13:**
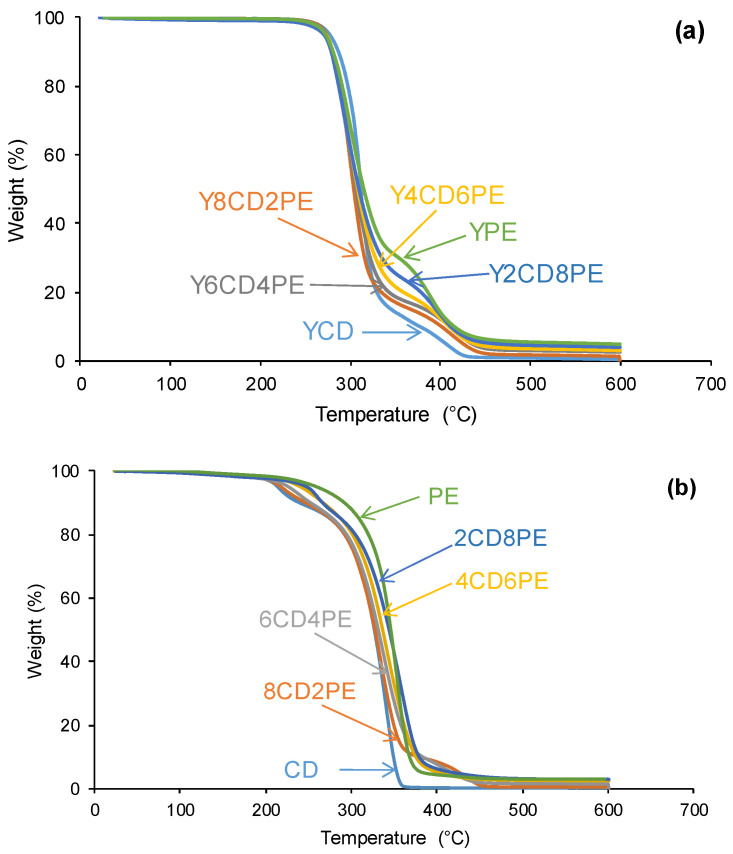
Variation of the weight loss as a function of the temperature of (**a**) PUs made with CD+PE blends and (**b**) CD+PE polyols blends.

**Figure 14:**
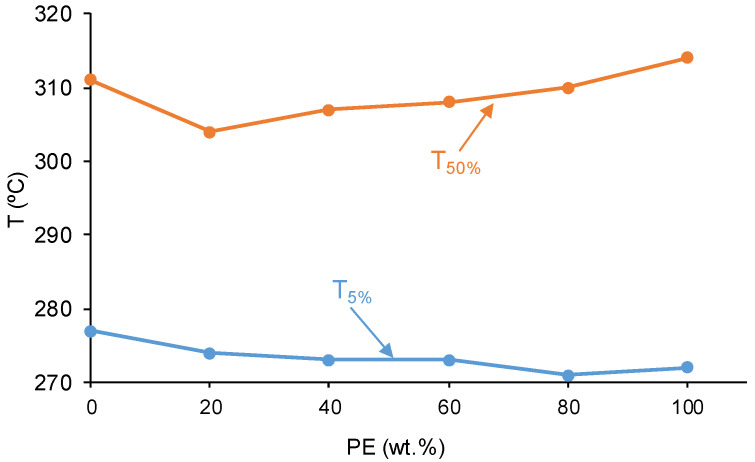
Variation in the temperatures at which 5% (T_5%_) and 50% (T_50%_) mass loss is produced in PUs made with CD+PE blends as a function of their PE content.

The thermal stabilities of PUs made with CD+PE blends are related to their structures and were assessed by TGA. Figure 13 shows two main thermal degradations in all PUs at about 300 °C (the most important) and 350 °C. Two thermal degradations are also evidenced in the polyols blends, one due to interactions between the carbonate groups at 210–260 °C and another at 300 °C (the most important) (Figure 13). The thermal degradation of the PUs at about 350 °C becomes more important by increasing their polyester soft segments content. The temperatures at which 5 (T_5%_) and 50 (T_50%_) wt.% mass are lost are indicative of the differences in the TGA curves of the PUs. Figure 14 shows that the T_5%_ value decreases and the T_50%_ value increases by increasing the polyester soft segments content in the PUs. The increase in T_50%_ value in the PUs by increasing their polyester soft segments content agrees well with the increase in their micro-phase separation. On the other hand, the T_50%_ value of Y8CD2PE is lower than the one of YCD, because of the intercalation of the polyester soft segments among the polycarbonate soft segments.

The derivatives of the TGA curves (DTGA curves) of the PUs better evidence the differences in the TGA curves (Figure S5 of Supplementary Material file). The DTGA curves of all PUs show two thermal degradations. YCD shows thermal degradations at 311 °C (polycarbonate soft segments) and 409 °C (hard segments), and YPE has thermal degradations at 300 °C (polyester soft segments) and 390 °C (hard segments). PUs made with CD+PE blends also show two thermal degradations at 296–300 °C and 397–416 °C (Table 8). The temperatures of degradation of the soft segments in PUs made with CD+PE blends are similar to the ones of YPE and smaller than the ones of YCD, and they do not vary by changing the polyester soft segments content. Thus, in Y8CD2PE, lower interactions between the polycarbonate soft segments are obtained by adding 20 wt.% PE only during synthesis. Furthermore, the weight loss from the thermal degradation of the soft segments in the PUs decreases from 84 to 77 wt.% by increasing their polyester soft segments contents, more noticeably in Y2CD8PE. On the other hand, in Y8CD2PE and Y6CD4PE, the hard segments degrade at 416–417 °C with a weight loss of 14 wt.% (Table 8). The increase in the PE content above 40 wt.% decreases the degradation temperature of the soft segments to 397 °C and increases the weight loss to 20 wt.%; again, this evidences the different structures in PUs made with more or less than 40 wt.% PE.

**Figure 15:**
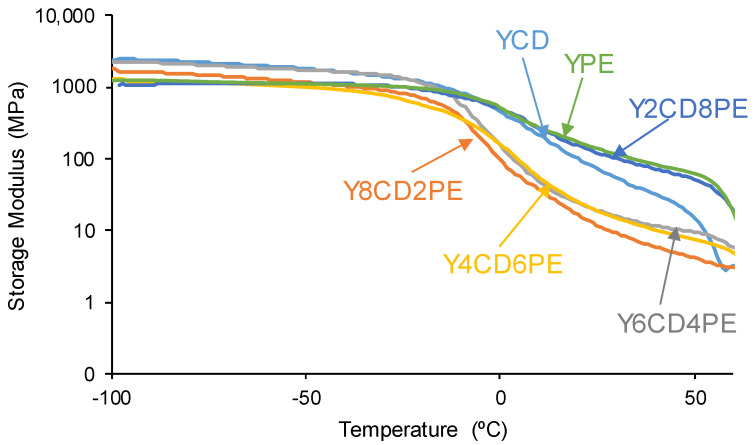
Variation of the storage (E’) moduli as a function of the temperature for PUs made with CD+PE blends. DMA experiments.

The structures of PUs made with CD+PE blends affect their rheological properties and viscoelasticity. According to the DMA curve of the variation in the storage modulus as a function of the temperature (Figure 15), at low temperatures (glassy region), the storage moduli of the PUs decrease by increasing their polyester soft segment content. Once the glass transition is reached, YPE and Y2CD8PE show the highest storage moduli and, thus, the mobility of their polymeric chains is restricted. However, the rest of the PUs made with CD+PE blends show significantly lower storage moduli, even lower than the one of YCD, and the mobility of their polymeric chains should be favored.

**Figure 16:**
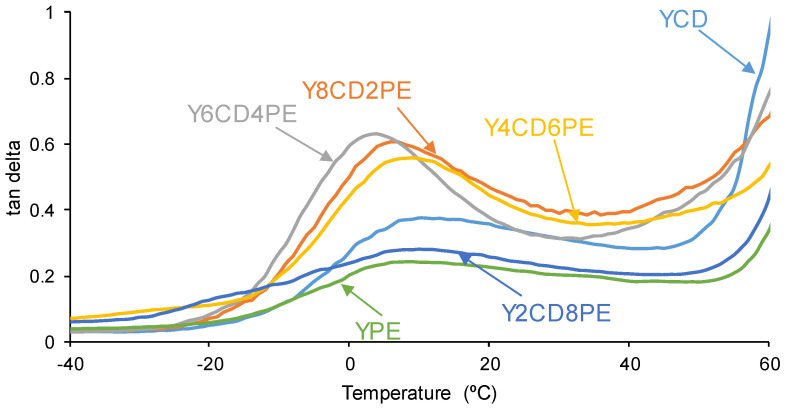
Variation of tan delta as a function of the temperature for PUs made with CD+PE blends. DMA experiments.

On the other hand, all PUs made with CD+PE blends show only one structural relaxation at 8–10 °C in the tan delta vs. temperature plots (Figure 16). The lowest tan delta values (0.24–0.28) correspond to YPE and Y2CD8PE, so they are dominantly elastic. Y8CD2PE, Y6CD4PE, and Y4CD6PE have tan delta values of 0.55–0.62, so they are more viscous and should show self-healing at 20 °C. However, even Y2CD8PE has a low tan delta value (0.28), the existence of some polycarbonate soft segments in its structure is sufficient to impart self-healing. Therefore, the self-healing property of PUs made with CD+PE blends depends not only on the mobility of the polymeric chains but also on the existence of polycarbonate soft segments.

### 3.4. Mechanical Properties of the PUs

The self-healing PUs should show adequate mechanical properties. YCD shows self-healing at 20 °C, but its mechanical properties are low (it is mainly elastomeric)—break strength: 590 kPa; elongation at break > 1119% (Table 9). However, YPE does not exhibit self-healing and is a stiff thermoplastic polymer—break strength: 3830 kPa; elongation at break: 50% (Table 9). It can be expected that the mechanical properties of the PUs made with CD+PE blends are better than the ones of YCD.

**Figure 17:**
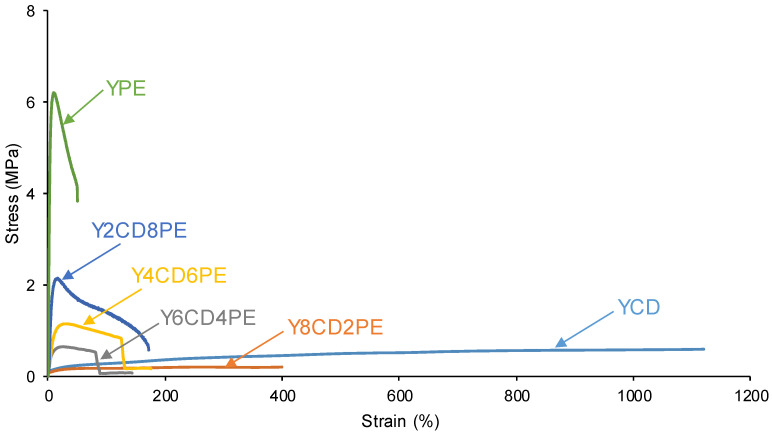
Stress–strain curves of the PUs made with CD+PE blends.

The stress–strain curves of the PUs made with CD+PE blends are shown in Figure 17. Y8CD2PE shows similar elastomeric properties to YCD (Table 9). However, the stress–strain curves of Y6CD4PE, Y4CD6PE, and Y2CD8PE show somewhat high Young moduli (90–320 kPa), a marked yield point, acceptable break strengths (490–790 kPa), and adequate elongation-at-break values (81–172%). Therefore, the mechanical properties of the PUs made with blends of polyols with 40 wt.% or more PE are acceptable, and they also show self-healing at 20 °C.

## 4. Discussion

YCD and all PUs made with CD+PE blends show self-healing at 20 °C. There are some previous studies on self-healing PUs made with polycarbonates [23,24,25,26], but none of them have shown fast self-healing at room temperature. Zhang et al. synthesized PUs that self-healed at 37 °C in 6 h, and the self-healing was ascribed to hydrogen bonding and mobile flexible short chains [26]. In addition, PUs that self-heal after heating at 120 °C for different times or at 80 °C for 40 s have been described [23,25]. From these studies, it seems clear that self-healing in PUs is favored by the mobility of the polymeric chains and the existence of hydrogen bonds between the hard segments. However, in the PUs of this study, the amount of hard segments is too small for justifying fast self-healing at 20 °C, and the interactions between polycarbonate soft segments themselves and with polyester soft segments seem to be the main driving force for self-healing.

Depending on the polyester soft segments content in the PUs, the self-healing time and kinetics of self-healing at 20 °C are different. Considering their self-healing time and kinetics of self-healing (Figure 3 and Figure 5), the PUs can be ranked into three different groups:

1. YCD and Y8CD2PE—The PUs made with only CD polyol and with 80 wt.% CD + 20 wt.% PE show short self-healing times (1.4–2.0 s) and fast kinetics of self-healing.
2. Y6CD4PE and Y4CD6PE—The PUs made with 40–60 wt.% CD + 60 − 40 wt.% PE show somewhat similar intermediate self-healing times (4.6–6.2 s) and intermediate kinetics of self-healing.
3. Y2CD8PE—The PU made with 20 wt.% CD + 80 wt.% PE shows a long self-healing time (20.3 s) and slow kinetics of self-healing.

The structures of these three groups of PUs are also different, and they can be related to their different self-healing abilities.

Y8CD2PE: The chemical structures of Y8CD2PE and YCD are almost similar, but Y8CD2PE has a lower percentage of free urethane/carbonate–carbonate interactions and a higher percentage of free carbonate species, in addition to higher micro-phase separation than YCD. Furthermore, Y8CD2PE shows a higher ∆c_p_ value in the glass transition of the soft segments, a more intense diffraction peak of the polycarbonate soft segments, and a new diffraction peak due to interactions between the polycarbonate and polyester soft segments; furthermore, the diffraction peaks of the polyester soft segments are absent. On the other hand, Y8CD2PE has significantly higher tan delta values and lower storage moduli than YCD.

**Figure 18:**
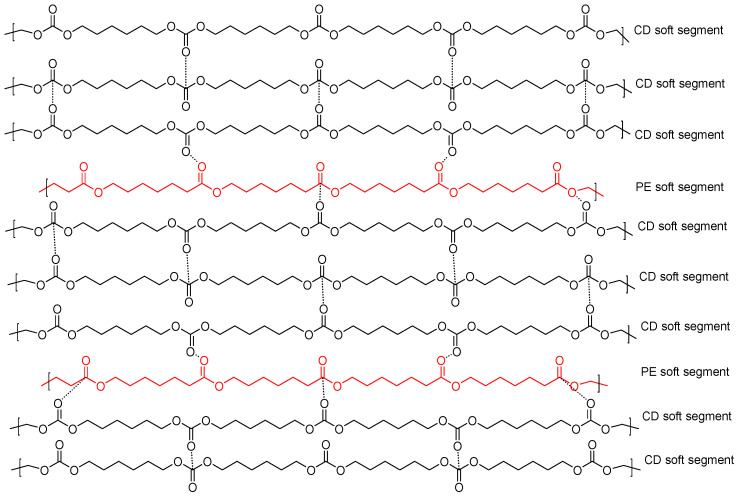
Potential interactions between carbonate groups in the PU made with 80 wt.% CD and 20 wt.% PE (Y8CD2PE).

These experimental findings evidence that the intercalation of a small amount of polyester soft segments among the polycarbonate soft segments decreases the interactions between them, leading to more net interactions in Y8CD2PE (Figure 18). As a consequence, the self-healing time (1.4–2.0 s) and kinetics of self-healing at 20 °C of YCD and Y8CD2PE must be very similar because, upon the application of stress, the rupture will be preferentially produced among the polycarbonate soft segments and the hydrogen bonds between urea/urethane and carbonate groups, and, upon stopping the stress, they would self-heal by restoring the polycarbonate soft segments interactions.

Y2CD8PE: Y2CD8PE shows a lower I_C=O_/I_OC(O)O_ ratio, lower percentage of free urethane/carbonate–carbonate interactions, and higher percentage of hydrogen-bonded urethane/ester–ester interactions, as well as somewhat higher micro-phase separation than YPE. Furthermore, Y2CD8PE shows a higher ∆c_p_ value in the glass transition of the soft segments and lower cold crystallization and melting enthalpies of the soft segments than YPE. On the other hand, Y2CD8PE shows a diffraction peak due to the interactions between the polyester and polycarbonate soft segments and a more intense diffraction peak of the polyester soft segments than YPE; additionally, the diffraction peaks of the polycarbonate soft segments are absent.

**Figure 19:**
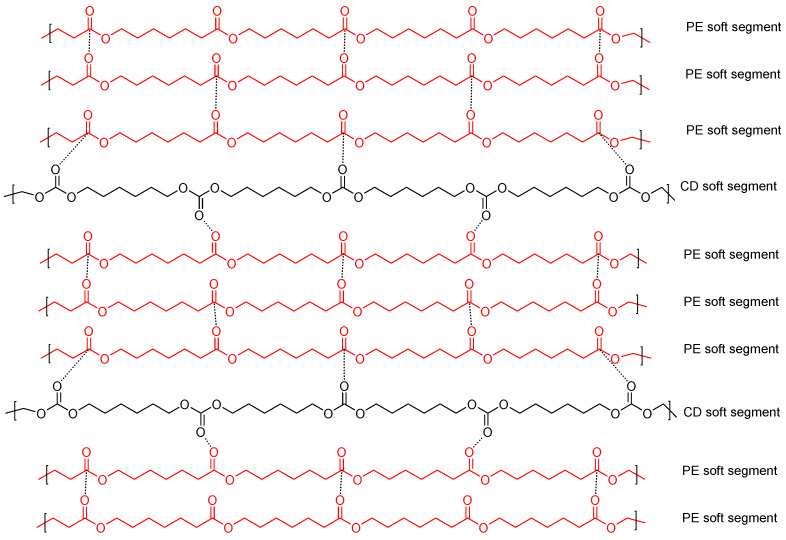
Potential interactions between carbonate groups in the PU made with 20 wt.% CD and 80 wt.% PE (Y2CD8PE).

Y2CD8PE shows self-healing at 20 °C, and YPE does not. Y2CD8PE has a longer self-healing time (20.3 s) and slower kinetics of self-healing than the other PUs made with CD+PE blends. Thus, the existence of a small amount of polycarbonate soft segments in Y2CD8PE is the main cause of its self-healing ability, even the restricted mobility of the polymeric chains (Y2CD8PE exhibits cold crystallization, high micro-phase separation, high storage moduli, and low tan delta values). Furthermore, the intercalation of the polycarbonate soft segments among the polyester soft segments causes the disruption of the crystallinity and the interactions between the polyester soft segments (Figure 19). Thus, upon the application of stress, a rupture will be produced between the polycarbonate and polyester soft segments and the hydrogen bonds between urea/urethane and carbonate groups, and, upon stopping the stress, Y2CD8PE would self-heal by restoring these interactions.

Y6CD4PE and Y4CD6PE: Both PUs have somewhat similar amounts of polycarbonate and polyester soft segments, so competition among soft segments of similar or different nature to interact with each other can be expected. Thus, Y4CD6PE and Y6CD4PE show similar I_C=O_/I_OC(O)O_ ratios (0.60–0.65), similar percentages of free urethane/carbonate–carbonate interactions (31–35%) and free carbonate (31%) species, similar high ∆c_p_ values in the glass transition of the soft segments, and similar tan delta values (0.55–0.62). However, Y4CD6PE shows a higher percentage of hydrogen-bonded urethane/ester–ester interactions, lower percentage of carbonate–ester species, higher micro-phase separation, and more intense diffractions peaks of the polyester soft segments and of the interactions between the polycarbonate and polyester soft segments than in Y6CD4PE. Therefore, the structural features of both polycarbonate and polyester soft segments appear in these PUs.

**Figure 20:**
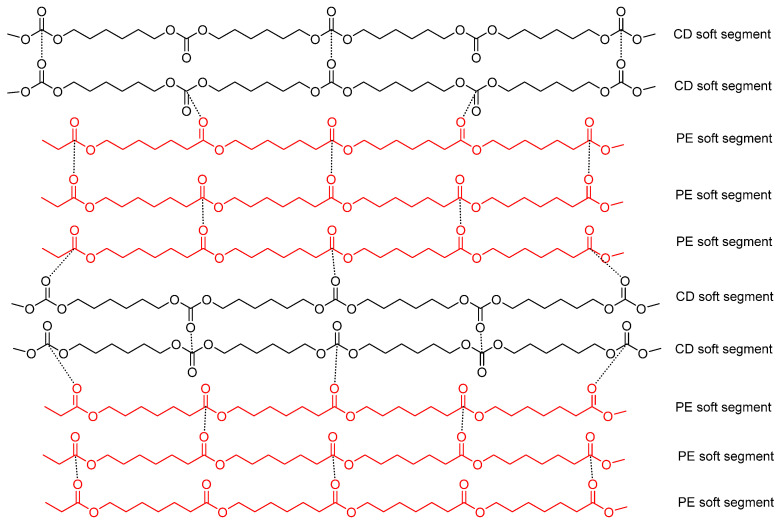
Potential interactions between carbonate groups in PU made with 40 wt.% CD and 60 wt.% PE (Y4CD6PE).

The self-healing times (4.6–6.2 s) and the kinetics of self-healing of Y4CD6PE and Y6CD4PE are somewhat similar, likely due to the competitive interactions between soft segments of similar or different nature (Figure 20). Thus, upon the application of stress, a rupture will be produced between the polycarbonate soft segments and between the polycarbonate and polyester soft segments, as well as among the hydrogen bonds between urea/urethane and carbonate groups. Upon stopping the stresses, Y4CD6PE and Y6CD4PE would self-heal by restoring these interactions.

## 5. Conclusions

All PUs made with CD+PE blends showed self-healing at 20 °C. The self-healing time was longer and the kinetics of self-healing was slower by decreasing the polycarbonate soft segments content. Furthermore, a small percentage of polycarbonate soft segments in the PU was sufficient to impart self-healing. The intrinsic self-healing at 20 °C in PUs made with CD+PE blends was ascribed to the existence of polycarbonate soft segments, the mobility of the polymeric chains, and the interactions between polycarbonate soft segments themselves and with polyester soft segments. Therefore, the self-healing of the PUs made with CD+PE blends was ascribed to physical interactions (dipole carbonate–carbonate, dipole ester–carbonate interactions, hydrogen bonds), and the previously proposed mechanisms of dynamic non-covalent exchange between polycarbonate soft segments was confirmed.

In Y6CD4PE and Y4CD6PE, the polyester soft segments–polycarbonate soft segments interactions were dominant, so they behaved differently than the PUs containing low amounts of polyester or polycarbonate soft segments. All PUs had free carbonate groups that allowed the movement of the polymeric chains and, at the same time, the polycarbonate soft segments became more aligned and the interactions between soft segments of a different chemical nature was favored. As the polyester soft segments content in the PU increased, the percentages of free urethane/urea and hydrogen-bonded urethane species increased, as well as the micro-phase separation; this caused longer self-healing times.

The structures of the PUs made with blends containing 20 wt.% CD or 20 wt.% PE were somewhat similar to the ones of the PUs made with only one polyol. On the other hand, the polyester soft segments–polycarbonate soft segments interactions decreased the percentage of free urethane groups, and this favored slower self-healing.

Y2CD8PE and YPE were the only PUs showing cold crystallization of the soft segments, and they exhibited high storage moduli and low tan delta values, in addition to similar micro-phase separation. However, the existence of a small amount of polycarbonate soft segments in Y2CD8PE was sufficient to disrupt the interactions between the polyester soft segments, and this PU exhibited self-healing at 20 °C.

Finally, the PUs made with blends containing 40 wt.% or more PE showed acceptable mechanical properties—somewhat high Young moduli, marked yield points, acceptable break strengths, and adequate elongation-at-break values.

## Figures and Tables

**Table 1:** Rate constants and regression values of the curves of self-healing at 20 °C of the PUs made with CD+PE blends (Figure 3).

PU	k (1/s)	R2
YCD	0.91	0.97
Y8CD2PE	0.68	0.92
Y6CD4PE	0.41	0.98
Y4CD6PE	0.28	0.98
Y2CD8PE	0.14	0.99

**Table 2:** Percentages of different carbonyl species of PUs made with CD+PE blends. Curve fitting of the C=O stretching region of the ATR-IR spectra.

Wavenumber (cm^−1^)	Percentage (%)						Assignment
	YCD	Y8CD2PE	Y6CD4PE	Y4CD6PE	Y2CD8PE	YPE	
1662–1674	9	5	3	5	4	8	Bonded urea
1693–1697	15	10	8	6	7	10	Free urea
1707–1716	14	12	10	17	23	26	Bonded urethane, ester–ester
1720–1722	-	13	13	10	11	-	Carbonate–ester
1730–1734	38	25	35	31	32	56	Carbonate–carbonate, Free C=O (ester), free urethane
1739–1744	24	35	31	31	23	-	Free C=O (carbonate)

**Table 3:** Parameters for the determination of micro-phase separation in PUs made with CD+PE blends: *f* = hard segment molar ratio; X_b_ = hydrogen-bonded urethane groups fraction; *W_h_* = fraction of the hard segment dispersed in the soft segment; *MP* = mixed-phase fraction; *SP* = soft-phase fraction; *HP* = hard-phase fraction.

PU	*f*	*X_b_*	*W_h_*	*MP*	*SP*	*HP*
YCD	0.22	0.23	0.18	0.04	0.82	0.18
Y8CD2PE	0.22	0.29	0.17	0.04	0.82	0.18
Y6CD4PE	0.22	0.19	0.19	0.04	0.82	0.18
Y4CD6PE	0.22	0.31	0.16	0.04	0.82	0.18
Y2CD8PE	0.22	0.37	0.15	0.03	0.81	0.19
YPE	0.23	0.27	0.18	0.04	0.81	0.19

**Table 4:** Chemical species on the surfaces of PUs made with CD+PE blends. XPS survey.

Species	Percentage (at.%)					
	YCD	Y8CD2PE	Y6CD4PE	Y4CD6PE	Y2CD8PE	YPE
C	76.4	75.5	79.9	72.5	73.9	75.4
O	23.2	23.4	19.1	25.7	24.4	22.8
N	0.4	1.1	1.0	1.8	1.7	1.8

**Table 5:** Chemical species on the PUs made with CD+PE blends surfaces. C1s photopeak. XPS experiments.

Species	Percentage (at.%)					
	YCD	Y8CD2PE	Y6CD4PE	Y4CD6PE	Y2CD8PE	YPE
C-C, C-H B.E. = 284.9 eV	84.1	77.3	75.9	67.1	68.8	69.2
C-O B.E. = 285.4–285.6 eV	6.6	3.2	3.3	3.9	2.9	2.6
C-N B.E. = 286.6–286.9 eV	7.1	14.6	14.3	20.7	20.3	19.1
C=O B.E. = 289.1–289.3 eV	1.5	3.4	5.3	5.9	6.2	9.1
O-(C=O)-O B.E. = 290.5–290.8 eV	0.7	1.5	1.2	2.4	1.8	-

**Table 6:** Thermal events obtained from the DSC curves of the PUs made with CD+PE blends. First heating run.

PU	T_g_ (°C)	Δc_p_ (J/g·°C)	T_c_ (°C)	ΔH_c_ (J/g)	T_m_ (°C)	ΔH_m_ (J/g)
YCD	−21	0.29	-	-	-	-
Y8CD2PE	−26	0.39	-	-	-	-
Y6CD4PE	−30	0.39	-	-	-	-
Y4CD6PE	−33	0.37	-	-	44	0.2
Y2CD8PE	−36	0.31	22	1	42	4
YPE	−40	0.35	21	4	42	7

**Table 7:** Glass transition temperatures obtained from the DSC curves of the PUs made with CD+PE blends. Second heating run.

PU	T_ss_ (°C)	T_hs_ (°C)
YCD	−18	236
Y8CD2PE	−24	233
Y6CD4PE	−29	238
Y4CD6PE	−31	235
Y2CD8PE	−38	240
YPE	−37	241

**Table 8:** Temperatures and weight losses of the thermal degradations of PUs made with CD+PE blends. DTGA experiments.

PU	1st Degradation		2nd Degradation	
	T_1_ (°C)	Weight Loss_1_ (%)	T_2_ (°C)	Weight Loss_2_ (%)
YCD	311	87	409	13
Y8CD2PE	300	84	416	14
Y6CD4PE	300	83	417	14
Y4CD6PE	299	81	406	16
Y2CD8PE	296	77	397	20
YPE	300	69	390	26

**Table 9:** Parameters obtained from the stress–strain curves of the PUs made with CD+PE blends.

PU	Young Modulus (kPa)	Yield Point		Break Point	
		σ_y_ (kPa)	ε_y_ (%)	σ_b_ (kPa)	ε_b_ (%)
YCD	60	-	-	590	˃1119
Y8CD2PE	90	-	-	200	398
Y6CD4PE	100	600	20	490	81
Y4CD6PE	90	1150	27	790	125
Y2CD8PE	320	2150	16	570	172
YPE	1680	6200	9	3830	50

## Data Availability

Data are contained within the article and Supplementary Materials.

## Supplementary Materials

The following supporting information can be downloaded at: https://www.mdpi.com/article/10.3390/polym16202881/s1. Figure S1. Fitting of the kinetics of self-healing at 20 °C of the PUs made with CD+PE blends to a first order kinetics equation; Figure S2. Curve fitting of the carbonyl stretching region of the ATR-IR spectra of some PUs; Figure S3. C1s photopeaks of some PUs. XPS experiments; Figure S4. DSC curves of the PUs made with CD+PE blends. Second heating run; Figure S5. Derivatives of the TGA curves of the PUs made with CD+PE blends. TGA experiments; Table S1. 2θ values and intensities of the main diffraction peaks of the PUs made with CD+PE blends. X-ray diffraction experiments. Video S1: Y4CD6PE self-healing.
