# Supplementary material for: Polyurethanes Synthesized with Blends of Polyester and Polycarbonate Polyols—New Evidence Supporting the Dynamic Non-Covalent Exchange Mechanism of Intrinsic Self-Healing at 20 °C
Source: Polymers (Basel). 2024 Oct 12;16(20):2881. doi: 10.3390/polym16202881 (PMC11511022; doi:10.3390/polym16202881)
Supplement: Supplementary file 1 [file polymers-16-02881-s001.zip › 2024-09-25 Supplementary Materials-PUs with CD+PE.pdf]

polymers

Supplementary materials

Polyurethanes synthesized with blends of polyester and polycarbonate polyols - New evidences supporting the dynamic non-covalent exchange mechanism of intrinsic self-healing at 20 °C

Yuliet Paez-Amieva, Noemí Mateo-Oliveras and José Miguel Martín-Martínez *

Adhesion and Adhesives Laboratory. University of Alicante, 03080 Alicante, Spain
* Correspondence: jm.martin@ua.es; Tel.: +34-965903977

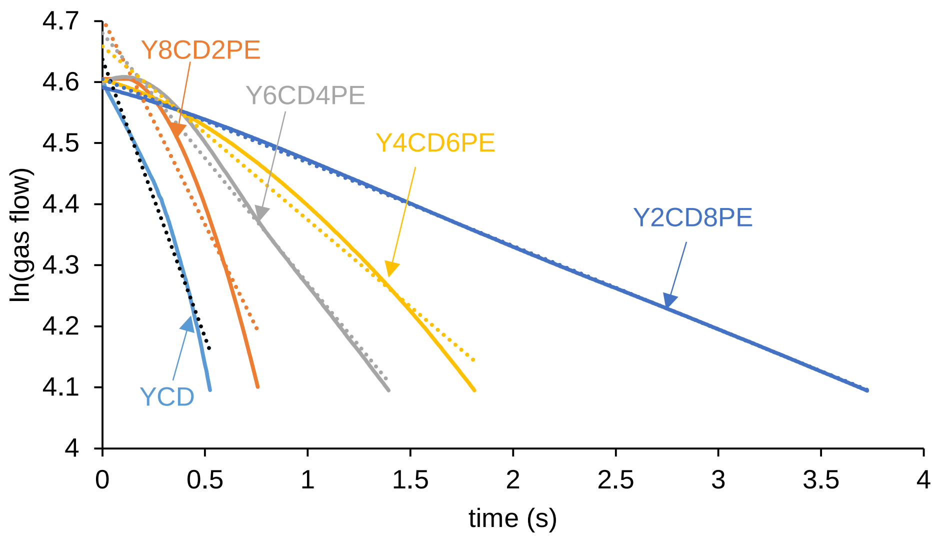

Figure S1. Fitting of the kinetics of self-healing at 20 °C of the PUs made with CD+PE blends to a first order kinetics equation.

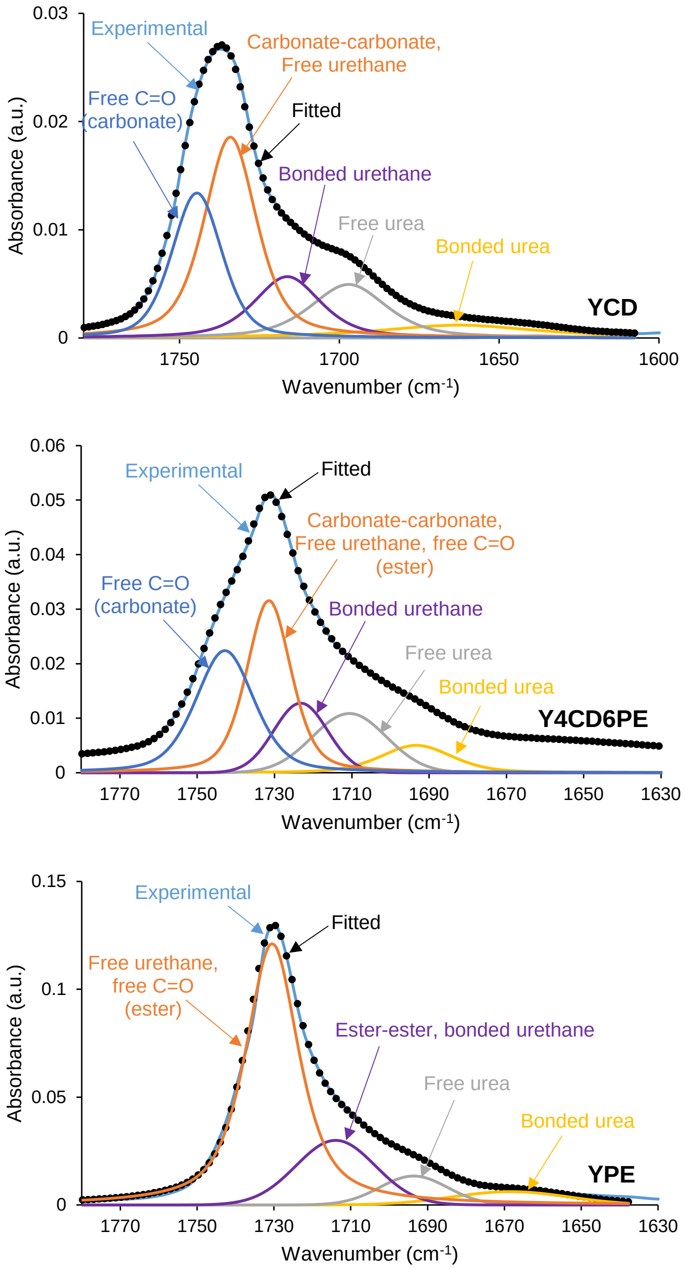

Figure S2. Curve fitting of the carbonyl stretching region of the ATR-IR spectra of some PUs.

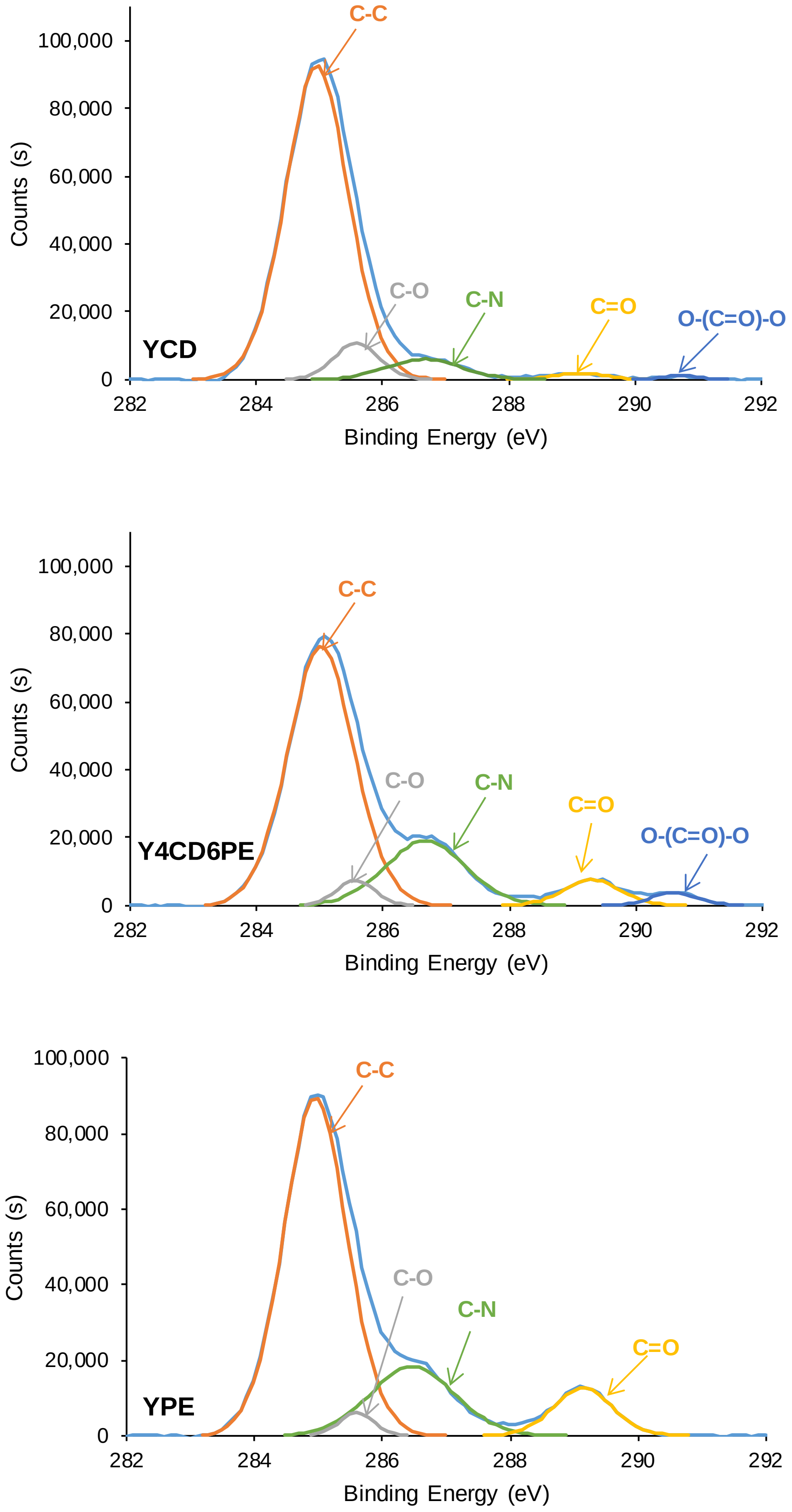

Figure S3. C1s photopeaks of some PUs. XPS experiments.

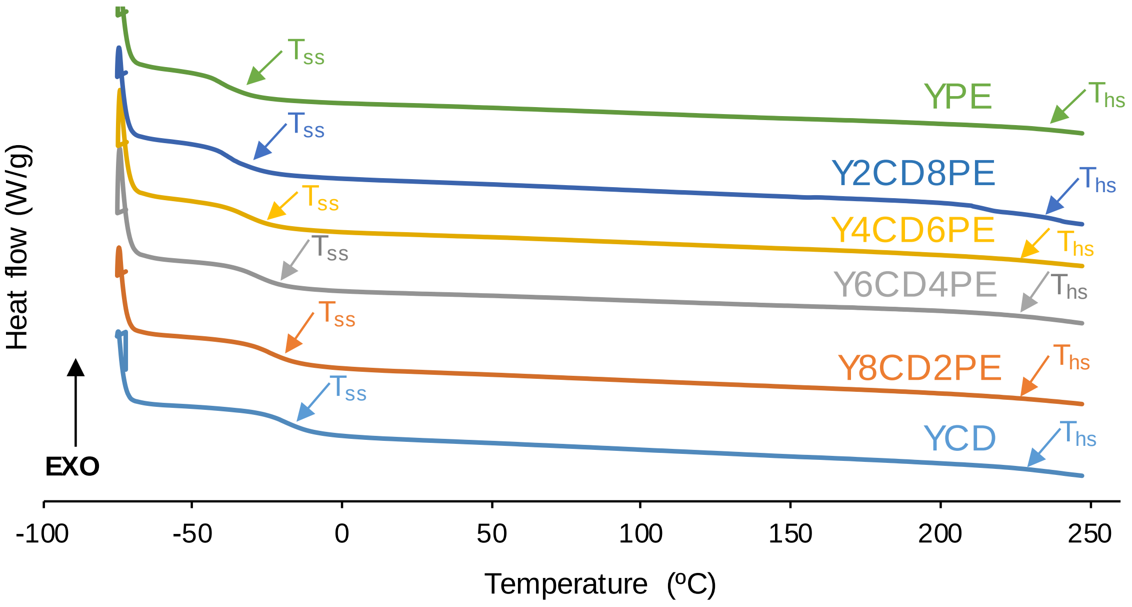

Figure S4. DSC curves of the PUs made with CD+PE blends. Second heating run.

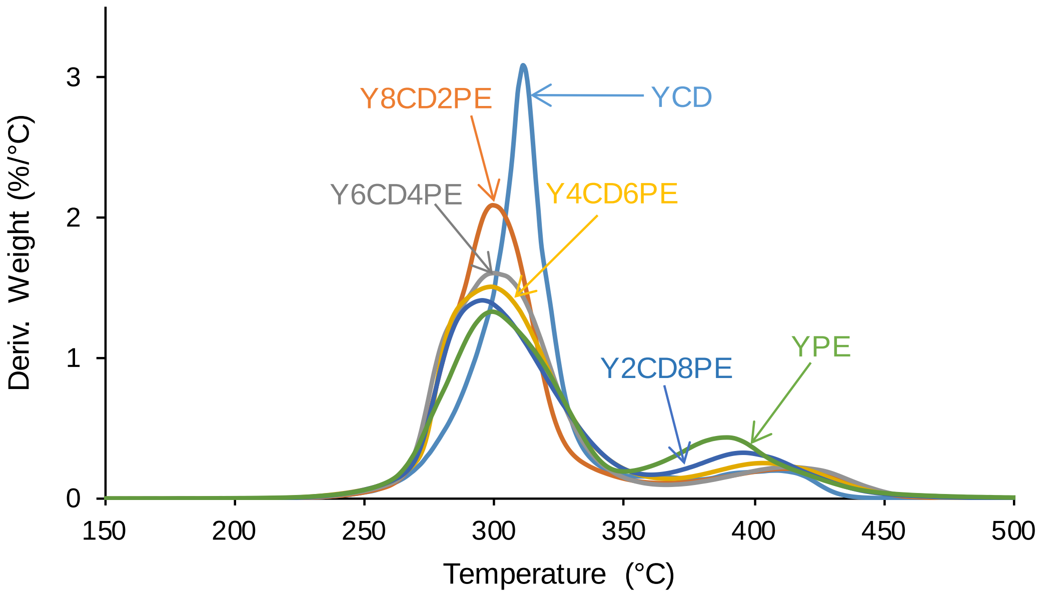

Figure S5. Derivatives of the TGA curves of the PUs made with CD+PE blends. TGA experiments.

Table S1. 2θ values and intensities of the main diffraction peaks of the PUs made with CD+PE blends. X-ray diffraction experiments.

PU	2θ (°)	Intensity (a.u.)	2θ (°)	Intensity (a.u.)	2θ (°)	Intensity (a.u.)
YCD	20.1	3186	-		-	
Y8CD2YPE	20.3	4456	-		21.8	3390
Y6CD4YPE	20.3	3853	21.4	4529	21.5	4550
Y4CD6YPE	20.1	4564	21.1	5345	21.8	5388
Y2CD8YPE	-		21.3	6988	21.7	7401
YPE	-		21.4	4182	-	

PU	2θ (°)	Intensity (a.u.)	2θ (°)	Intensity (a.u.)	2θ (°)	Intensity (a.u.)
YCD	-		23.4	1677	-	
Y8CD2YPE	22.5	3063	23.5	4100	-	
Y6CD4YPE	22.4	3954	23.5	3259	-	
Y4CD6YPE	22.6	3383	23.4	3102	24.2	2866
Y2CD8YPE	22.4	4825	-		24.2	2793
YPE	22.1	2092	-		23.8	859
